# Gene selection algorithm by combining reliefF and mRMR

**DOI:** 10.1186/1471-2164-9-S2-S27

**Published:** 2008-09-16

**Authors:** Yi Zhang, Chris Ding, Tao Li

**Affiliations:** 1 School of Computer Science, Florida International University, 11200 SW 8th Street, Miami, FL, 33199, USA; 2 Department of Computer Science and Engineering, University of Texas at Arlington, 416 Yates Street, Arlington, TX, 76019, USA

## Abstract

**Background:**

Gene expression data usually contains a large number of genes, but a small number of samples. Feature selection for gene expression data aims at finding a set of genes that best discriminate biological samples of different types. In this paper, we present a two-stage selection algorithm by combining ReliefF and mRMR: In the first stage, ReliefF is applied to find a candidate gene set; In the second stage, mRMR method is applied to directly and explicitly reduce redundancy for selecting a compact yet effective gene subset from the candidate set.

**Results:**

We perform comprehensive experiments to compare the mRMR-ReliefF selection algorithm with ReliefF, mRMR and other feature selection methods using two classifiers as SVM and Naive Bayes, on seven different datasets. And we also provide all source codes and datasets for sharing with others.

**Conclusion:**

The experimental results show that the mRMR-ReliefF gene selection algorithm is very effective.

## Background

Gene expression refers to the level of production of protein molecules defined by a gene. Monitoring of gene expression is one of the most fundamental approach in genetics and molecular biology. The standard technique for measuring gene expression is to measure the mRNA instead of proteins, because mRNA sequences hybridize with their complementary RNA or DNA sequences while this property lacks in proteins. The DNA arrays, pioneered in [1,2], are novel technologies that are designed to measure gene expression of tens of thousands of genes in a single experiment. The ability of measuring gene expression for a very large number of genes, covering the entire genome for some small organisms, raises the issue of characterizing cells in terms of gene expression, that is, using gene expression to determine the fate and functions of the cells. The most fundamental of the characterization problem is that of identifying a set of genes and its expression patterns that either characterize a certain cell state or predict a certain cell state in the future [3].

When the expression dataset contains multiple classes, the problem of classifying samples according to their gene expression becomes much more challenging, especially when the number of classes exceeds five [4]. Moreover, the special characteristics of expression data adds more challenge to the classification problem. Expression data usually contains a large number of genes (in thousands) and a small number of experiments (in dozens). In machine learning terminology, these datasets are usually of very high dimensions with undersized samples. In microarray data analysis, many gene selection methods have been proposed to reduce the data dimensionality [5].

Gene selection aims to find a set of genes that best discriminate biological samples of different types. The selected genes are "biomarkers", and they form "marker panel" for analysis. In general, two types of gene selection methods have been studied in the literature: filter methods [6] and wrapper methods [7]. As pointed out in [8], the essential differences between the two methods are:

(1) that a wrapper method makes use of the algorithm that will be used to build the final classifier while a filter method does not, and

(2) that a wrapper method uses cross validation to compare the performance of the final classifier and searches for an optimal subset while a filter method uses simple statistics computed from the empirical distribution to select attribute subset.

Wrapper methods could perform better but would require much more computational costs than filter methods. Most gene selection schemes are based on binary discrimination using rank-based schemes [9], such as information gain, which reduces the entropy of the class variables given the selected attributes. In expression data, many gene groups interact closely and gene interactions are important biologically and may contribute to class distinctions. However, the majority of the rank-based schemes assume the conditional independence of the attributes given the target variable and are thus not effective for problems involving much feature interaction [10].

In this paper, we present a two-stage selection algorithm by combining ReliefF [10] and mRMR [11]. ReliefF, a general and successful attribute estimator, is able to effectively provide quality estimates of attributes in problems with dependencies between attributes. mRMR (minimal-redundancy-maximal-relevance) method selects genes that have the highest relevance with the target class and are also maximally dissimilar to each other. mRMR is computationally expensive. The integration of ReliefF and mRMR thus leads to an effective gene selection scheme. In the first stage, ReliefF is applied to find a candidate gene set. This filters out many unimportant genes and reduces the computational load for mRMR. In the second stage, mRMR method is applied to directly and explicitly reduce redundancy and select a compact yet effective gene subset from the candidate set. We perform comprehensive experiments to compare the mRMR-ReliefF selection algorithm with ReliefF, mRMR and other feature selection methods using two classifiers on seven different datasets. The experimental results show that the mRMR-ReliefF gene selection is very effective.

## Result and discussion

In this section, we perform comprehensive experiments to compare the mRMR-ReliefF selection algorithm with ReliefF, mRMR and other feature selection methods using two classifiers (Support Vector Machine (SVM) and Naive Bayes (NB)) on seven different datasets.

### Datasets description

The datasets and their characteristics are summarized in Table 1.

**Table 1:** The dataset description.

Dataset	# Samples	# Genes	# Classes
ALL	248	12558	6
ARR	420	278	2
GCM	198	16063	14
HBC	22	3226	3
LYM	62	4026	3
MLL	72	12582	3
NCI60	60	1123	9

• **ALL**: The ALL dataset [12] is a dataset that covers six subtypes of acute lymphoblastic leukemia: BCR (15), E2A (27), Hyperdip (64), MLL (20), T (43), and TEL (79). Here the numbers in the parentheses are the numbers of samples. The dataset is available at [13].

• **ARR**: The Arrhythmia (ARR) dataset contains 420 samples and 278 features with two classes [14].

• **GCM**: The GCM dataset [15] consists of 198 human tumor samples of fifteen types. breast (12), prostate (14), lung (12), colorectal (12), lymphoma (22), bladder (11), melanoma (10), uterus (10), leukemia (10), renal (11), pancreas (11), ovary (120), mesothelioma (11), CNS (20), and MET (9). The prediction accuracy of 78% is reported in [15] using one-versus-the rest SVM with all the genes.

• **HBC**: The HBC dataset consists of 22 hereditary breast cancer samples and was first studied in [16]. The dataset has three classes and can be downloaded at [17].

• **LYM**: The Lymphoma dataset is a dataset of the three most prevalent adult lymphoid malignancies and available at [18] and it was first studied in [19].

• **MLL**: The MLL-leukemia dataset consists of three classes and can be downloaded at [20].

• **NCI60**: The NCI60 dataset was first studied in [21]. cDNA microarrays were used to examine the variation in gene expression among the 60 cell lines from the National Center Institute's anticancer drug screen. The dataset spans nine classes and can be downloaded at [17,22].

Note that in these datasets, the samples in each class is generally small, and unevenly distributed. This, together with the large number of classes, especially for NCI60, GCM, makes the classification task more complex.

### Compare ReliefF, mRMR and mRMR-ReliefF algorithm

**Figure 1:**
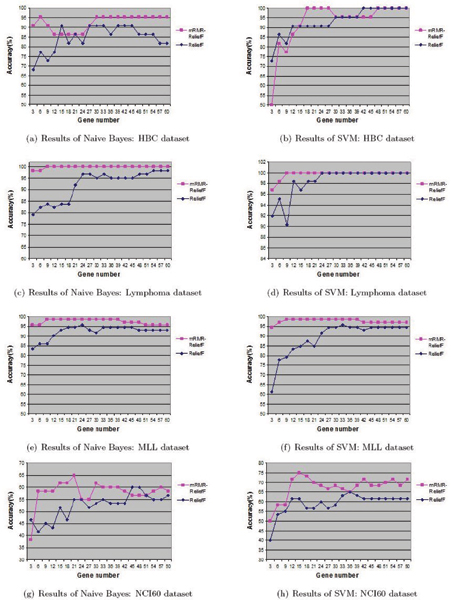
**Comparison of ReliefF and mRMR-ReliefF algorithms I**. This figure describes the two classifications (SVM and NB) results using 3 to 60 selected genes, for HBC, Lymphoma, MLL, and NCI60 datasets. From this figure, it is easy to know that in the same number of selected genes, the performance of mRMR-ReliefF algorithm is obviously better than ReliefF algorithm.

**Figure 2:**
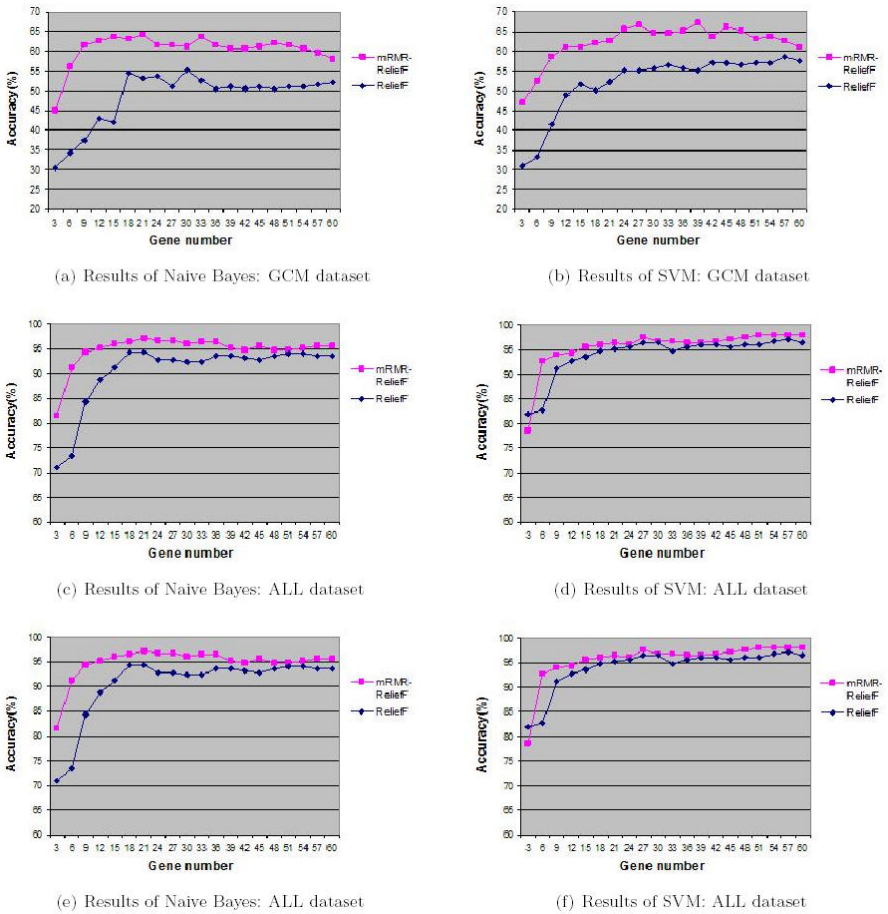
**Comparison of ReliefF and mRMR-ReliefF algorithms II**. This figure describes the two classifications (SVM and NB) results using 3 to 60 selected genes, for GCM, ALL, and ARR datasets. From this figure, it is easy to know that in the same number of selected genes, the performance of mRMR-ReliefF algorithm is obviously better than ReliefF algorithm.

First we compare the mRMR-ReliefF algorithm with ReliefF and mRMR. We perform our comparisons using SVM and NB classifiers on the seven datasets. Both SVM and NB have been widely used in previous studies. Figure 1 and Figure 2 show the classification accuracy results as a function of the number of selected genes on the seven datasets respectively. In addition, because of mRMR is computationally expensive, using the program provided in [11], we could not obtain results for several datasets with a large number of genes, e.g., ALL and GCM. Thus in the figures, we only include the accuracy values for ReliefF and the mRMR-ReliefF algorithm and these values are all obtained via 10-fold cross validation.

Table 2 presents the detail of the accuracy values of applying SVM and NB classification on the top 30 selected genes, for some unavailable results which can not be computed by mRMR, we note them as "-". From the above comparative study, we observe that:

• The performance of mRMR algorithm is pulled down by its expensive computational cost, and it can not fulfill gene selection on the database with large features using the limited memory.

• Relief algorithm is not stable enough when only a small number of genes are selected. And when the number of selected genes is greater than 30, the variations of classification performance of both ReliefF and mRMR-ReliefF algorithms are generally small.

• The mRMR-ReliefF selection algorithm leads to significantly improved class predictions. With the same number of selected genes, the gene set obtained by the mRMR-ReliefF selection is more representative of the target class, therefore leading to better class prediction or generalization property.

**Table 2:** The comparisons in ReliefF, mRMR and mRMR-ReliefF algorithms (gene number = 30)

**Feature Selection Method**	**Classifier**	**ALL**	**ARR**	**LYM**	**HBC**	**NCI60**	**MLL**	**GCM**
ReliefF	SVM	96.37%	79.29%	100%	95.45%	58.33%	94.44%	55.25%
	Naive Bayes	92.34%	75%	95.16%	90.91%	53.33%	91.67%	55.56%
mRMR	SVM	-	75.35%	100%	95.45%	53.33%	-	-
	Naive Bayes	-	73.21%	97.33%	87.51%	51.20%	-	-
mRMR-ReliefF	SVM	96.77%	81.43%	100%	95.45%	68.33%	98.61%	64.65%
	Naive Bayes	95.97%	79.05%	100%	95.45%	61.67%	98.61%	61.11%

### Comparison with other methods

We also compare our mRMR-ReliefF selection algorithm with other gene selection algorithms, including Max-Relevance, Information Gain, Sum Minority, Twoing Rule, F-statistic [23], and GSNR [24]. Table 3 presents the classification accuracy comparison using SVM and NB classifier based on the selected genes using these six feature selection methods, when the number of selected gene is 30. From Table 3, we observe that:

**Table 3:** The comparisons in seven gene selection methods (gene number = 30).

**Feature Selection Method**	**Classifier**	**ALL**	**ARR**	**LYM**	**HBC**	**NCI60**	**MLL**	**GCM**
No feature sel	SVM	91.94%	51.04%	95.16%	77.27%	63.33%	97.22%	51.52%
	Naive Bayes	85.23%	49.57%	95.04%	70.11%	45.22%	93.13%	40.33%
mRMR-ReliefF	SVM	96.77%	81.43%	100%	95.45%	68.33%	98.61%	64.65%
	Naive Bayes	95.97%	79.05%	100%	95.45%	61.67%	98.61%	61.11%
Maxrel	SVM	89.11%	74.53%	100%	72.73%	51.67%	77.78%	60.61%
	Naive Bayes	88.71%	73.49%	100%	63.64%	48.33%	80.56%	46.97%
Information Gain	SVM	97.58%	80.13%	98.39%	100%	61.67%	98.67%	46.67%
	Naive Bayes	92.74%	77.21%	93.55%	86.38%	60%	97.22%	47.47%
Sum Minority	SVM	93.95%	76.42%	98.39%	95.45%	55%	90.28%	55.05%
	Naive Bayes	91.13%	74.32%	95.16%	81.82%	46.67%	91.67%	49.49%
Twoing Rule	SVM	96.77%	79.37%	98.39%	90.91%	61.67%	97.22%	45.96%
	Naive Bayes	90.32%	72.19%	93.55%	86.36%	45%	95.83%	46.46%
F-statistic	SVM	97.17%	67.12%	96.77%	90.91%	63.33%	77.22%	39.10%
	Naive Bayes	80.27%	71.55%	98.52%	85.41%	60.15%	80.13%	39.81%
GSNR	SVM	93.18%	77.24%	100%	95.45%	63.37%	90.25%	40.74%
	Naive Bayes	90.11%	70.43%	100%	85.65%	58.25%	87.22%	39.81%

This table shows the classification results based on the 30 genes, which are selected from 7 different datasets using seven feature selection methods, named mRMR-ReliefF, Maxrel, information gain, sum minority, twoing rule, F-statistic, GSNR.

• Gene selection improves class prediction. Note that the accuracy of SVM using feature selection generally outperforms that without feature selection. This implies that feature selection can effectively reduce the insignificant dimensions and noise to improve classification accuracy.

• The mRMR-ReliefF algorithm is shown to achieve better performance comparing with other gene selection algorithms on almost all datasets. The experimental comparisons demonstrate the effectiveness of the integration of ReliefF and mRMR.

• ReliefF achieves good performance on most of the data sets. Although its performance is not always as good as that of the mRMR-ReliefF algorithm. It outperforms mRMR, Maxrel, Sum Minority and partially wins information gain, twoing rule.

• Only a small number of genes are needed for classification purpose. In our experiments, the variations of the classification accuracy are small when the number of selected genes is greater than 30.

### Software package

We have developed a software package for the above experiments, which includes: 1) The source codes for four feature selection algorithms including ReliefF, F-statistic, GNSR, and Relief-mRMR; 2) A MATLAB interface for Rankgene1.1 [5] which contains another eight feature selection measures; 3) A MATLAB interface for two well-known classification tools (e.g., LIBSVM and WEKA); 4) Programs for converting data formats; 5) The collection of all datasets used in the experiments. We hope it is a useful tool in gene expression analysis and feature selection.

This package and all datasets can be downloaded from  All codes are implemented and tested in Matlab 7.0 and can be integrated into the Toolbox by adding its path to MATLAB search path.

#### Data structure and translation

**Figure 3:**
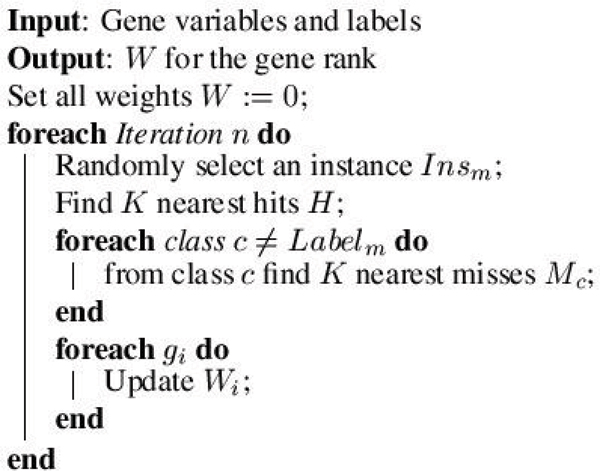
Description of ReliefF algorithm.

This package supports consistent data formats. Each gene dataset is formatted as a MATLAB data structure file(.mat), in which a class label vector corresponds to a gene array. For any algorithm, the input is a .mat file, and the output is an index vector for the selected genes. Furthermore, a utility is provided for converting the data from .csv file to .mat file. The command line is as follows.

csvtomat(*Filename*)

where *Filename *is the name of .csv file. In the .csv file, the first column is the class label, the rest are gene variables. For .mat file, its structure can be shown as Figure 3:

We also provide the function to convert .mat file to .csv file as:

mattocsv(*X*, *y*, *Filename*)

where *X*, *y *are the matrix defined in .mat file and *Filename *is the .csv file as output file.

#### Implementation of gene selection algorithms

The command list to perform different gene selection algorithms is shown in Table 4, where *X *is a gene array, *y *is a class label vector, and *Topn *is the number of selected genes in current algorithm. For ReliefF function, *n *is the number of iterations, *K *is the number of neighbors to be selected, and *typed *is the data type; for the Rankgene function, and *T *is the method index which can be referenced in rankgene1.1.

**Table 4:** MATLAB Command List For Gene Selection.

**Algorithm Description**	**Command Line**
ReliefF	*W *= reliefF(*X*, *y*, *n*, *K*, *typed*, *Topn*)
F-statistic	*W *= Ftest(*X*, *y*, *Topn*)
GNSR	*W *= Gsnr(*X*, *y*, *Topn*)
ReliefF-mRMR	*W *= rm(*X*, *y*, *n*, *K*, *Topn*)
Rankgene	*W *= rankgene(*X*, *y*, *T*, *Topn*)

This table shows MATLAB commands for the feature selection algorithms, which are ReliefF, F-statistic, GNSR, ReliefF-mRMR, and all algorithms included in Rankgene.

#### Assistant tools for classification

To compare the performance of the gene selection algorithms, we also include two popular classification tools in this software package, which are the existing MATLAB version for LIBSVM [25] and a MATLAB Interface for WEKA [26]. For LIBSVM, there is already a ready-to-run plug-in for MATLAB. And we implement the function for calling WEKA. The command line for calling WEKA is shown as follows.

$$\begin{array}{c} \text{mattocsv}(X,y,Filename)\\ Accuracy=\text{wekaclassifier}(Filename,Classifier) \end{array}$$

where *Filename *is the name of the output .csv file, *X *is a gene array, *y *is a label vector, and *Classifier *is the parameter for classification method, such as Naive Bayes and J4.5 tree.

## Conclusion

In this paper, we present an mRMR-ReliefF selection algorithm by combining ReliefF and mRMR. ReliefF is able to effectively provide quality estimates of attributes in problems with dependencies between attributes and mRMR method selects genes that have the highest relevance with the target class and are also maximally dissimilar to each other. The integration of ReliefF and mRMR thus leads to an effective gene selection scheme: In the first stage, ReliefF is applied to find a candidate gene set; In the second stage, mRMR is applied to select a compact yet effective gene subset from the candidate set.

Comprehensive experiments are conducted to compare the mRMR-ReliefF selection algorithm with ReliefF, mRMR and other feature selection methods using two classifiers on seven different datasets. The experimental results show that the mRMR-ReliefF gene selection is very effective. In addition, we also developed a software package to help other researches explore gene expression.

## Methods

In this part, firstly, ReliefF and mRMR algorithms are discussed, then mRMR-ReliefF selection algorithm is presented, and finally, other six gene selection algorithms used to compare with our mRMR-ReliefF algorithm are introduced.

### ReliefF

ReliefF is a simple yet efficient procedure to estimate the quality of attributes in problems with strong dependencies between attributes [10]. In practice, ReliefF is usually applied in data pre-processing as a feature subset selection method.

**Figure 4:**
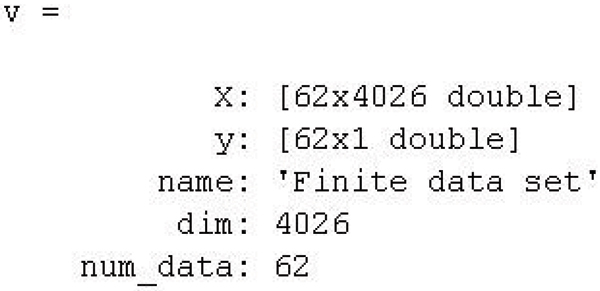
**The data structure description for software package**. *X *is the gene array with 62 genes and 4026 expression variables. *y *is the label for each gene.

The key idea of the ReliefF is to estimate the quality of genes according to how well their values distinguish between instances that are near to each other. Given a randomly selected instance *Ins*_*m *_from class *L*, ReliefF searches for *K *of its nearest neighbors from the same class called nearest hits *H*, and also *K *nearest neighbors from each of the different classes, called nearest misses *M*. It then updates the quality estimation *W*_*i *_for gene *i *based on their values for *Ins*_*m*_, *H*, *M*. If instance *Ins*_*m *_and those in *H *have different values on gene *i*, then the quality estimation *W*_*i *_is decreased. On the other hand, if instance *Ins*_*m *_and those in *M *have different values on the the gene *i*, then *W*_*i *_is increased. The whole process is repeated *n *times which is set by users. The algorithm is shown in Figure 4 and updating *W*_*i *_can use Equation 1:

(1) $$W_{i}=W_{i}-\frac{\sum_{k=1}^{K}D_{H}}{n\cdot K}+\sum_{c=1}^{C-1}P_{c}\cdot \frac{\sum_{k=1}^{K}D_{M_{c}}}{n\cdot K}$$

where *n*_*c *_is the number of instances in class *c*, *D*_*H *_(or $D_{M_{c}}$) is the sum of distance between the selected instance and each *H *(or *M*_*c*_), *P*_*c *_is the prior probability of class *c*.

Detailed discussions on ReliefF can be found in [10] and recently, it was shown that ReliefF is an on-line solution to a convex optimization problem, maximizing a margin-based algorithm [27].

### mRMR

The mRMR (minimum redundancy maximum relevance) method [11] selects genes that have the highest relevance with the target class and are also minimally redundant, i.e., selects genes that are maximally dissimilar to each other. Given g_*i *_which represents the gene *i*, and the class label *c*, their mutual information is defined in terms of their frequencies of appearances *p*(*g*_*i*_), *p*(*c*), and *p*(*g*_*i*_, *c*) as follows.

(2) $$I(g_{i},c)=\int p(g_{i},c)\ln\frac{p(g_{i},c)}{p(g_{i})p(c)}dg_{i}dc$$

The Maximum-Relevance method selects the top *m *genes in the descent order of *I*(*g*_*i*_, *c*), i.e. the best *m *individual features correlated to the class labels.

(3) $$\underset{S}{\max}\frac{1}{\left|S\right|}\sum_{g_{i}\in S}I(g_{i};c)$$

Although we can choose the top individual genes using Maximum-Relevance algorithm, it has been recognized that "the *m *best features are not the best *m *features" since the correlations among those top features may also be high [28]. In order to remove the redundancy among features, a Minimum-Redundancy criteria is introduced

(4) $$\underset{S}{\min}\frac{1}{{\left|S\right|}^{2}}\sum_{g_{i},g_{j}\in S}I(g_{i},g_{j})$$

where mutual information between each pair of genes is taken into consideration. The minimum-redundancy maximum-relevance (mRMR) feature selection framework combines both optimization criteria of Eqs.(3, 4).

A sequential incremental algorithm to solve the simultaneous optimizations of optimization criteria of Eqs.(3, 4) is given as the following. Suppose set *G *represents the set of genes and we already have *S*_*m*-1_, the feature set with *m*-1 genes. Then the task is to select the *m*-th feature from the set {*G *- *S*_*m*-1_}. This feature is selected by maximizing the *single-variable relevance minus redundancy *function

(5) $$\underset{g_{j}\in G-S_{m-1}}{\max}[I(g_{i};c)-\frac{1}{m-1}\sum_{g_{i}\in S_{m-1}}I(g_{j};g_{i})]$$

The *m*-th feature can also be selected by maximizing the *single-variable relevance divided-by redundancy *function

(6) $$\underset{g_{j}\in G-S_{m-1}}{\max}[I(g_{i};c)/\frac{1}{m-1}\sum_{g_{i}\in S_{m-1}}I(g_{j};g_{i})]$$

### mRMR-ReliefF algorithm

As we mentioned before, ReliefF is a general and successful attribute estimator and is able to effectively provide quality estimates of attributes in problems with dependencies between attributes. However, ReliefF does not explicitly reduce the redundancy in selected genes. mRMR selects genes that have the highest relevance with the target class and are also maximally dissimilar to each other. However, mRMR is computationally expensive. For example, using the mRMR program provided in [11], we could not obtain results for several datasets with a large number of genes, e.g., ALL and GCM. The integration of ReliefF and mRMR thus leads to an effective gene selection scheme.

We can view the *quality estimation W*_*i *_in ReliefF as maximizing the relevance score. Thus we can view the standard ReliefF algorithm as maximizing the relevance score:

(7) $$\underset{S}{\max}\frac{1}{\left|S\right|}\sum_{g_{i}\in S}W_{i}$$

Thus our mRMR-ReliefF algorithm selection criteria becomes

(8) $$\underset{g_{j}\in G-S_{m-1}}{\max}W_{i}-\frac{1}{m-1}\sum_{g_{i}\in S_{m-1}}\left|C(g_{j},g_{i})\right|$$

or

(9) $$\underset{g_{j}\in G-S_{m-1}}{\max}W_{i}/\frac{1}{m-1}\sum_{g_{i}\in S_{m-1}}\left|C(g_{j},g_{i})\right|$$

where C(*g*_*j*_, *g*_*i*_) is the Pearson correlation coefficient.

Our mRMR-ReliefF algorithm works as follows: In the first stage, ReliefF is applied to find a candidate gene set. This filters out many unimportant genes and reduces the computational load for mRMR. In the second stage, mRMR method is applied to directly and explicitly reduce redundancy and select a compact yet effective gene subset from the candidate set.

In our experiments, ReliefF is first used to choose 150 genes as the candidate set. from the all gene data. mRMR algorithm is then applied to select the final subset.

### Other gene selection algorithms

In this part, we introduce six other gene selection algorithms which are mentioned in the chapter of "Result and discussion", which are named Max-Relevance, Information Gain, Sum Minority, Twoing Rule, F-statistic [23], and GSNR [24]. These methods have been reported in previous work. The first four methods have been used either in machine learning (information gain) or in statistical learning theory (twoing rule and sum minority), and all of them measure the effectiveness of a feature by evaluating the strength of class prediction when the prediction is made by splitting it into two regions, the high region and the low region, by considering all possible split points [5]. More detailed descriptions on these methods can be found in [5].

F-statistic is chosen to score the relevance between the genes and the classification variable. The F-statistic of gene *i *in *C *classes has the following form [23]:

(10) $$W_{i}=\frac{\sum_{c=1}^{C}n_{c}\cdot \left(\bar{g_{ic}}-\bar{g_{i}}\right)/\left(C-1\right)}{\sum_{c=1}^{C}\left\{(n_{c}-1\right)[\sum_{i=1}^{n_{c}}{\left(g_{jic}-\bar{g_{ic}}\right)}^{2}/n_{c}]/(n-C)\}}$$

where *C *is the number of classes, $\bar{g_{i}}$ is the mean of gene *i *variables, *n*_*c *_is the number of samples in class *c*, $\bar{g_{ic}}$ is the mean of gene *i *in class *c*, and *g*_*jic *_is sample *j *in gene *i *value in class *c*.

As to GSNR, it has been proposed and used in [24]. GSNR is a measure of the ratio between inter-group and intra-group variations. Higher GSNR values indicate higher discrimination power for the gene. The GSNR value for gene *i *is given by:

(11) $$W_{i}=\frac{\sum_{c=1}^{C}|\bar{g_{jc}}-\sum_{c=1}^{C}\bar{g_{jc}}/C|/C}{\sum_{i=1}^{C}\sum_{i=1}^{n_{c}}|g_{jic}-\bar{g_{ic}}|/n_{c}}$$

Both F-statistic and GSNR select *m *genes in the descent order of *W*_*i*_, and the best subset of genes is satisfied the following description:

(12) $$\underset{S}{\max}\frac{1}{\left|S\right|}\sum_{g_{i}\in S}W_{i}$$

## Competing interests

T. Li is partially supported by NSF CAREER Award IIS-0546280 and NIH/NIGMS S06 GM008205. C. Ding is partially supported by a University of Texas STAR Award.

## Authors' contributions

T. Li and C. Ding initialized the idea and supervised the project. Y. Zhang implemented the algorithms, developed the software, performed experimental comparisons, and built the website. All authors have read and approved the manuscript.
